# Advance Care Planning Conversations in Pediatric Patients with Refractory Oncologic Disease

**DOI:** 10.3390/children12040479

**Published:** 2025-04-08

**Authors:** Aqsa Khan, Ajay Gupta, Andy Liu, Ali H. Ahmad

**Affiliations:** 1 Pediatric Critical Care Fellowship, Department of Pediatrics, Nicklaus Children’s Hospital, Miami, FL 33155, USA; aqsa.khan@nicklaushealth.org; 2 Pediatric Oncology, Roswell Park Comprehensive Cancer Center, Jacobs School of Medicine and Biomedical Sciences, University of Buffalo, Buffalo, NY 14203, USA; ajay.gupta@roswellpark.org; 3 Division of Critical Care, Department of Pediatrics, Georgetown University Medical Center, Washington, DC 20007, USA; 4 Section of Critical Care, Department of Pediatrics, MD Anderson Cancer Center, The University of Texas, Houston, TX 77030, USA; ahahmad@mdanderson.org

**Keywords:** advanced care planning, advanced directives, pediatric palliative care, end-of-life care, pediatric oncology

## Abstract

Advance care planning (ACP) involves longitudinal communication between the patient and physician to explore the patient’s wishes and goals while relaying accurate diagnostic and prognostic information to support informed and shared medical decision-making. In pediatrics, it also uniquely involves the parents or legal guardians as the primary medical decision-makers. ACP ideally leads to the implementation of advanced directives (ADs) and can be a difficult concept to discuss with pediatric patients and families with refractory oncologic disease, given the distinctive burdens that accompany this population. Many obstacles can delay the initiation of these conversations with these families, although existing literature supports beginning ACP conversations at the time of initial diagnosis and treatment. Parents or legal guardians often serve as the sole decision maker for pediatric patients but there has also been a shift in the literature to include children/adolescents in conversations regarding ADs and other aspects of end-of-life (EOL) care, an essential aspect of patient-centered ACP. This guidance is unfortunately not often translated into clinical practice. In this review, we aim to define and discuss the current status, obstacles, and benefits surrounding early initiation of ACP conversations with children or adolescents with advanced cancer and their families. We also discuss how physicians and the medical team, including pediatric palliative care, can increase the degree of pediatric patient involvement in ACP and EOL discussions, as developmentally appropriate, and mitigate delays in discussing ACP with these families and patients.

## 1. Introduction

The 5-year survival rate of pediatric cancers has improved to surpass 80% in developed countries [1] but remains one of the leading causes of death in pediatric patients, although the mortality differs by country [2,3]. Pediatric patients, defined in this review as younger than 21 years old, with refractory or treatment-resistant cancer face unique challenges as an understudied population [4]. They often have limited treatment options despite general advances in the pediatric oncology field [4,5]. In the absence of standard of care treatment options, the burden of therapeutic decision-making falls upon the caregiver(s) [6,7]. Patients with advanced cancer also face an increased likelihood of death, which adds additional stress to medical decision-making [8]. Life-prolonging therapies, in contrast to curative treatments, can complicate the disease trajectory, further intensifying the decision-making challenges faced by the family [9]. It becomes imperative to begin the conversations about advance care planning (ACP) early and throughout the entire course of illness to allow patients and families the time needed to process and make decisions before a potential medical crisis occurs.

ACP is described as (1) discussing and exploring the family and child/adolescent’s wishes, values, and goals, and (2) providing clear, comprehensive information about diagnosis, prognosis, and treatment options (including the uncertainty or lack of options if applicable) [10,11,12,13,14]. As the patient’s trajectory advances, ACP includes consistent reframing of goals and desires in the patient’s best interest by directly involving patients and families in these crucial conversations, with inclusion of pediatric patients as appropriate to their developmental age in these conversations [15,16]. It aims to support informed healthcare decision-making that aligns with these explored desires and is an ongoing process subject to change given an evolving prognosis and changes in the wishes of the patient and family [10,11,12,13,14]. Furthermore, the discussions themselves should be documented by the physician, with updates as the patient and/or family’s desires and goals change. Advance directives (ADs) and related medical orders should also be documented and updated accordingly [9]. Pediatric palliative care, defined as the active holistic care of a pediatric patient with advanced cancer in this setting, can help with this kind of care and documentation [17]. It not only encompasses those nearing end-of-life (EOL) but also aims to improve the quality of life for both the patient and their family throughout their care journey [17]. This involves working hand-in-hand with the pediatric oncologist, often at diagnosis for patients of advanced cancer, to assist with quality of life issues that can include pain, nausea, fatigue, etc.

In this review, we aim to define and discuss the current status, obstacles, and benefits surrounding early initiation of ACP conversations with children or adolescents with advanced cancer and their families. We also discuss how physicians and the medical team, including pediatric palliative care, can increase the degree of pediatric patient involvement in ACP and EOL discussions, as developmentally appropriate, and mitigate delays in discussing ACP with these patients and families.

## 2. Pediatric Development and ACP

Traditionally and legally, pediatric ACP focused on parents’ desires for their child and relied on the parent to be the patient’s advocate. However, there has been an academic shift to proposing more opportunities for children and adolescents to express their own goals and wishes about their care by including them—within their capacity and capability—in discussions surrounding medical decision-making and ACP [15,16]. In addition, pediatric patients should be respected by physicians and the medical team as autonomous individuals despite their lack of legal rights in medical decision-making [11,13,16]. Since 1995, the American Academy of Pediatrics (AAP) has recommended for assent to be obtained from pediatric patients, and if it cannot be provided or is not appropriate, then the patient should be given control of what they can choose according to their ability [16]. Additional international guidance from the World Health Organization and other international groups have echoed these recommendations [18,19]. Unique to pediatrics, the development, understanding, and capacity of the patient will be dynamic throughout the sometimes multi-year course of illness, which will subsequently influence their level of participation in decision-making [15,16]. Physicians also should be aware that pediatric patients may have capacity in certain areas but may lack it for more complex discussions, and so communication needs to be adjusted accordingly [16]. Also, developmental stage and calendar age do not always correlate in children and adolescents; battling with a severe illness can further impact the patient’s developmental age. Physicians should continuously reassess their patients’ ability to participate in various aspects of ACP discussions, as this can serve as an initial step in shifting from parent-centered to patient-centered care within clinical practice.

Younger children (less than 5 years) have been associated with later palliative care referrals [20]. Interestingly, there has been a bimodal association noted between age of child with cancer and the intensity of EOL treatment they receive [21]. Children less than 5 years of age and adolescents 15 to 21 years of age were associated with higher levels of EOL care [21], but it is unclear if this was in line with the patients’ and/or families’ wishes. Adult patients with advanced or terminal cancer typically choose less intense or life-extending care [21,22,23], although it is unknown if a similar perspective is shared by pediatric oncology patients. Some studies have shown that when a patient’s wishes and quality of life are factored into parent’s decision-making, it often causes a shift in therapy to palliation over cancer-directed therapy [24,25]. ACP, including ADs, has been associated with less intense EOL care for children with cancer [24,26,27].

Most adolescents do not differ from adults in their capacity to make informed treatment decisions, and their understanding of death is no less mature [16,28]. For example, one study found patients 10 to 20 years of age with advanced cancer were able to participate in complex EOL discussions and comprehend the multifactorial nature of their decisions and its subsequent effects on the near and far future [29]. They also exhibited a greater extent of altruism in their decisions than expected per current developmental theories by being willing to enroll in Phase 1 clinical trials to benefit others, even with the potential for toxicity [29]. Nonetheless, adolescents may lack social or emotional maturity, causing them to be prone to risk-taking or easily influenced by their current emotional state, which may widely fluctuate day-to-day or with new developments in their medical disease [30,31]. If the peer influence and emotional arousal can be minimized, older adolescents (above 14 years of age) exhibit similar maturity levels as adults [32]. Those factors may be difficult to ascertain but maintaining adequate physician-patient rapport may aid in including the patient in these discussions.

The majority of adolescents favored discussing ACP from the time of diagnosis or first illness and not when they are critically ill [28,33]. This offers patients an early opportunity to explore their wishes and desires and brings to the forefront the importance of their own desires in navigating the course of their illness. Unfortunately, these data have not evenly translated into clinical practice and pediatric patients are not always involved in ACP and EOL decision-making [34,35,36,37]. One qualitative study of audio recordings of conversations between a physician, the parents, and the patient found that 29 of 35 pediatric cancer patients (age range 2.5–17.5 years, median 10.2 years) were at least partially present for physician-family discussions of uncertainties at time of cancer progression, including topics regarding prognosis or response to treatment, diagnostic uncertainty, logistical uncertainty, appropriateness of treatments, and acute and long-term toxicities. However, in the 35 clinical encounters recorded, the patient initiated less than 2% (10/489) of statements of uncertainty with regards to the aforementioned topics [37]. While there were a small number of patients who were likely not developmentally able to contribute statements, this study nevertheless highlights the stark discrepancy in the ultimate goal of including children/adolescents in ACP discussions and the current reality.

## 3. Familial Readiness for Pediatric ACP

Hope is a common theme for parents of children/adolescents with refractory cancer and is a significant influence in parental decision-making [24,38]. Even when parents recognize death is a likely possibility for their child, some literature suggests that it does not cause a shift from cancer-directed therapy towards palliation, as there is still hope in desiring for more time and/or wanting to try every alternative therapy [5]. Parents of children with advanced cancer often make medical decisions for their children based on these overly hopeful expectations [39,40]. This can lead to increased levels of medical EOL care, suffering, and symptom burden [12]. Parents often perceive more hope for a cure even if not verbalized by the medical team [41]. While it behooves physicians to deliver accurate prognostic information as clearly and as early as possible to parents of a child with advanced cancer, physicians often delay and avoid ACP conversations in children with little hopes of cure [42].

Current literature suggests parents of children with advanced cancer may lack awareness of their child’s desires in some facets of EOL planning, although not for lack of prioritizing their child’s health. Studies have demonstrated that families often understood their children’s desires in terms of wanting honest answers from their physician and understanding treatment choices, but often poorly understood desires for dying a natural death, for initiation of EOL conversations, or for making EOL decisions if their disease were to worsen (such as being off machines that extend life if already dying) [27,28]. One study of 80 families reported that only 39% of families knew their adolescent cancer patient wanted to talk about their diagnosis early, although 86% of adolescents stated wanting to do so [28]. Another study of 131 bereaved caregivers had a disproportionately greater number of caregivers talk about prognosis or the concept of death with their children and demonstrated that almost half of the children asked about dying or spoke about their own death [43].

Parents of children with advanced cancer often pursue cancer-directed therapies along with supportive care, even when there is no realistic hope of cure [24,39,44]. Some parents who chose further treatment cited the biggest factor in their decision was feeling compelled to continue cancer-directed therapy but rarely discussed level of care in their decision-making process [25]. As a result, some families may have initial apprehension over the establishment of ADs, including do-not-resuscitate (DNR) documentation, in that it may reduce the level or quality of medical care their children receive or be perceived as “giving up”. However, one retrospective chart review at a single pediatric cancer center showed that multiple medical interventions were continued in most cases, except for chemotherapy, which was continued in a third of cases [45]. Of note, documentation of a DNR order was, on average, 34 days prior to patient’s death, which may explain shifting of goals of care. There were also infrequent life-prolonging interventions reported as well, such as surgery, continuous renal replacement therapy, and vasopressor usage, even in cases of incurable illness. This suggests physicians may use multiple treatment options despite advancement or severity of disease and, while DNR status may suggest a shift in care goals, life-prolonging interventions can concurrently occur.

Fortunately, taking the time to assess the family’s goals has shown to reduce EOL decisional regret by both the patient and the family [10,46]. Listening to parental hesitations and concerns without judgment may mitigate the intense pressure felt by parents making such impactful decisions. The most vital factors in EOL decision-making according to parents include feeling their child’s needs are heard, symptom management, and goal-concordant care [47,48]. As a result, parents who were more prepared for EOL scenarios reported less perceived suffering of the child [9,49]. Including the patient in ACP and EOL discussions has also been shown to shift medical decision-making by the parents to a more shared decision-making model and has led to overall congruence and higher quality of life [24,26,27]. One study found parental openness in communication near the time of diagnosis or relapse in children with advanced cancer was predictive of their child’s future emotional adjustment [8]. This finding was not replicated in the non-advanced cancer comparison group, emphasizing the unique importance of early involvement of pediatric patients with advanced cancer in ACP discussions. Parents who acknowledge the poor prognosis of their child’s cancer are less likely to pursue cancer-directed therapy near EOL [40,50]. Pediatric cancer patients and families who engage in ACP conversations with their physicians are more likely to have resuscitation orders and ADs in place at time of child’s death [10,33], thus ideally assisting with EOL decisions. As a result, it often becomes the physician’s responsibility to be aware of the many aspects ACP can encompass and facilitate discussions addressing these areas.

Involving patients in EOL conversations has also shown to be less distressing for parents despite their initial apprehension as it decreases the likelihood of parental decisional regret, decreases anxiety and depression scores reported at follow-ups, and improves grief outcomes even years after their child passing of cancer [51,52,53,54]. In one study of 429 bereaved parents, 34% of parents talked about death with their children diagnosed with cancer and none of them regretted doing so at follow-up [51]. Of those that chose not to do so, 27% did regret not having done so and had higher levels of anxiety at follow-up. In another study of 131 bereaved caregivers, parents who felt unprepared for their child’s medical problems and emotional needs had distress and prolonged grief symptoms [49]. The most common type of distress was over not involving their child in EOL discussions. This highlights the complexity in determining the extent to include a child in discussions involving EOL care and/or death, which varies depending on the individual patient’s age, temperament, illness course, sociocultural background, as well as other factors.

In some countries, enrollment in hospice care does not preclude continuation of life-prolonging treatment, thereby relieving families from having to choose between the two and potentially improving quality of EOL care and mitigating caregiver grief [55]. One study showed children with advanced cancer enrolled in concurrent hospice care were classified as receiving moderate or high intensity care (none in the low intensity category) and significantly more services than other children with chronic conditions, suggesting that the unique burdens of oncologic disease were continuing to be addressed throughout the disease course even while enrolled in hospice [56]. Another study that compared 226 20-year-olds with cancer enrolled in concurrent hospice care to those without cancer enrolled in concurrent hospice care found that the first group sought treatment for symptoms more often from nonhospice providers (35.4% vs. 14.0%) [57]. Concurrent care can also offer the benefit of greater ease of transition between cancer-directed therapy and palliation if patient/family’s wishes were to shift. It ideally allows for all-encompassing patient care while minimizing patient and caregiver burden.

## 4. Timing of Introduction of Pediatric ACP

It is generally recommended that the topic of advanced care should be introduced to families of children with life-limiting conditions “early”, but there is no set definition for when the initial conversation should occur [11,58]. Some literature recommends beginning the process either when diagnosis has been confirmed or prognosis has been deemed poor [14,58]. Another proposal is to start ACP conversations at time of treatment [52]. This could potentially emphasize the concept of parallel planning, which is allowing one to plan for the child’s full potential in life and death simultaneously [11]. However, some clinicians delay ACP at this time out of concern for parents requiring more time to process the initial news before commencing a conversation about advanced care [59]. Some societies recommend triggers or changes in patient’s illness or understanding of illness to be the start of a conversation of revisiting goals of care with parents, acknowledging the non-linear process ACP can take [9].

While the benefits of early ACP conversations are known and endorsed in pediatric oncology [12], uncertainty among physicians regarding when to approach ACP conversations often delays initiation of these conversations [60] and it has often been found to be lacking in clinical practice [14,61]. When they do occur, they often occur late in the disease process [12,14,20,61,62]. One study recorded 141 conversations between physicians and families at serial follow-ups of patients with high-risk cancer (defined as survival less than 50%) over 24 months and found over 75% of conversations around ACP or palliative care occurred after clear evidence of disease progression [12]. Additionally, only 4.8% of discussions after cancer had progressed included topics of palliative care or ACP, suggesting the limited amount of time spent on these conversations. Other considerations noted to delay initiation of ACP have included intercultural differences [14,15,58,63], time constraints in office [14], difficulty in dealing with changing demands of developing child [15,36,59], legality of dealing with adolescents [52], uncertainty over who should initiate conversations of ACP [59], and lack of familiarity with AD documentation [59].

Parents often prefer earlier ACP-related conversations, as it allows families time for EOL planning and implementing decisions that would better align with the patient’s goals [10,12,20,64,65]. However, the uncertainty in disease progression or prognosis that can be seen in children with refractory or advanced cancer also makes it difficult to know when to approach topics of advanced care, and is likely a factor in why these conversations often occur after progression of disease [10,14,15,37,61]. One study in pediatric patients with recently progressed cancer found that physicians were the ones to initiate most conversations around uncertainty of disease/prognosis and did so usually by providing more information instead of acknowledgement and support [37]. Diagnostic uncertainty and logistical uncertainty were only discussed in 10% of 489 clinical encounters recorded [37]. There is often limited information to provide in cases of disease/prognostic uncertainty, which may be why these conversations occur infrequently.

Introducing ACP early and continuously with patients and families can ideally lead to greater congruency and decreased emotional burden of decision-making amongst patients, families, and medical teams. Physicians can worry that bringing up topics of ACP or asking parents to sign an AD can add additional emotional stress during an already difficult time or suggest the medical team is giving up hope [9,14,52,58,60,66]. They also fear parental reactions when asking them to sign an AD [9,14,58,66]. This is a valid concern, as parents report making EOL decisions to be the most difficult part of their child’s cancer experience [6]. However, a multicenter study demonstrated that prognostic disclosure to families of pediatric oncology patients was not associated with anxiety, depression, or decreased hope [67]. Additionally, a prospective qualitative study showed that in parents of children with relapsed or refractory cancer, only a minority of parents (4/32 pairs) were upset by the clinician communication about prognosis and most did not blame the clinician but rather the situation [65].

## 5. Interventions and Perspectives to Improve Pediatric ACP Adoption

Acknowledging the uncertainty of the illness trajectory and prognosis, even if there is limited medical information to provide, could serve as an introduction to ACP. Addressing disease uncertainty has been shown to increase parental hope as they feel better prepared prognostically and for their child’s last days [65]. It could also ideally reinforce the notion that this will be continuously revisited due to its uncertain nature to encourage open and ongoing conversations. Parents of children with advanced cancer report ongoing conversations with the physician to be critical in making EOL decisions for their child and ensuring higher quality of care [46,68]. Those parents who reported receiving high quality information were also strongly associated with lower levels of decisional regret [69].

Multiple trials of intervention tools to involve adolescents with advanced or poor-prognosis cancer directly in ACP conversations allowed for increased congruence between adolescents and their parents for EOL decision-making [26,27]. These studies included three weekly sessions using standardized templates to guide documentation and discussions to explore patient’s and family’s wishes regarding various aspects of ACP and subsequent documentation [26,27]. It also led to more patients having documentation for medical wishes and/or orders in place prior to time of death [26]. One trial found that even at 12 months follow-up, there was high overlap with parent and adolescent wishes, and parents were more likely to support the adolescent’s decisions [33]. This was not supported at 18 months follow-up, suggesting that more frequent follow-up visits may be indicated to maintain congruence [33], especially considering cases with a quickly evolving prognosis. Standardized templates did not increase family emotional distress or strain and led to improved relationships between parents and adolescents [65,70]. Parents did not lose hope even in cases with poor prognoses and were able to acknowledge the discrepancy between diagnosis and internal hope for further treatment or cure [65,71]. ACP has also been shown to lead to less anxiety and depression for adolescents at follow-up as well [27]. Overall, discussing ACP in a templated manner with a pediatric patient in an age-appropriate manner can help guide informed decision-making by parents and physicians and also improve quality of EOL care [52]. Further research is needed to explore the shift in pediatric patient’s wishes along their illness course in refractory or advanced cancer.

Clearly communicating an accurate prognosis in cases of poor or uncertain outcomes can help families pivot their hope to hope applicable for a patient’s current condition, such as hope for better quality of remaining time [46]. Parents do often hope for a cure even in cases of incurable cancer, which should be respected, and physicians who discuss the reality of prognosis early and accurately can encourage hope concurrently for present-day circumstances and the future, demonstrating that hope and realism can coexist. Children with advanced cancer versus non-advanced cancer specifically benefit from parental openness early in the disease course, showing fewer depressive symptoms [8]. Focusing on the patient with ACP and future treatment planning also benefits the child to understand and decrease frustration by “combining the inner and outer worlds” [51]. By directly involving the child in the aspects of decision-making they can appropriately partake in, patient-centered ACP can tangibly demonstrate patient’s emerging autonomy and allow them to feel they have some degree of control during a time where many things are not in their control. Respecting the patient’s autonomy often increases medical compliance and allows them to be more accepting of treatment and medical care as well [16].

Parents may prefer to be given resources or guidelines on how to talk about the concept of death or poor prognoses with their children rather the physician speaking about these topics with their child [43]. This could be another way to shift the trajectory of patient care from parent-centered to patient-centered. Given the emotional complexity in navigating their child’s diagnosis of advanced cancer, parents should not be presumed to carry the burden of assuming their child’s desires for EOL care if there is an opportunity for the child to express their own opinions. With patients who lack the cognitive ability to understand their diagnosis or what EOL entails, parent-centered care becomes the default. Even in these cases, physicians should take care to emphasize decision-making to be on behalf of the patient and for what is best for the patient. In cases where the child or adolescent can be involved then they should be relied on as their own speaker with parents and medical team serving as advocates for their wishes. By providing parents with the resources and skills necessary to discuss these emotionally difficult concepts with their child from the initial stage of diagnosis and treatment and continuing to reassess parental readiness in approaching ACP discussions with their child, physicians can partner with caregivers to incorporate patient-centered ACP into clinical practice. Patient-centered care does require more responsibilities and duties on behalf of the physician but can lead to greater benefits as depicted in Table S1.

More clinician training is required. Some pediatric oncologists have acknowledged discomfort with their own skill level and lack of specific training in approaching ACP as factors in delaying EOL conversations [9,14,59,72]. This may have to do with the lack of formal or standardized education on ACP, palliative care, and ethics for pediatric oncologists [42,46]. Pediatric trainees often learn EOL or palliative care learning through observing other medical physicians, which then becomes based on personal experiences and exposures [46]. One study of 77 pediatric oncologists showed that 50% reported undertaking palliative care training but only 33% completed a dedicated palliative care rotation [62]. Studies have shown that providing education to physicians on how to conduct ACP conversations prior to implementing standardized templates in guiding discussions for ACP with patient and family was essential to ACP confidence in and success of interventions [11,53]. Some oncologists may also believe that palliative care subspecialists will initiate those discussions and thus avoid bringing up aspects of ACP themselves [59]. However, one survey showed that parents prefer an intensive care unit (ICU) or subspecialist physician to begin the initial conversation about ACP [10]. In line with these wishes, given the typically longer duration of the parent-physician relationship in oncology, the pediatric oncologist would be the ideal initiator of this conversation. Parent-physician relationship is crucial to providing higher EOL quality care to pediatric patients with refractory cancer [46]; therefore, it is essential for pediatric oncologists to have the skills to introduce, address, and reassess ACP with parents. It is also important to initiate those ongoing conversations early, as the parent-physician relationship is a significant factor in influencing a parent’s EOL decision-making [5,24,73]. This is particularly important in scenarios where palliative care resources or providers are limited [62].

## 6. Palliative Care and “A Good Death”

Even in pediatric cancer treatment centers when palliative care subspecialists are more abundant, there is no standardized timing of consult and they are often involved late (defined as less than 30 days before death or after disease progression) in patients with advanced cancer [20,61]. Physicians may be concerned that involving palliative care may precipitate the discussion of ADs and EOL care prior to parents or child being ready, but a retrospective chart review showed early consultation did not involve EOL discussion in the initial visits [74]. Instead, palliative care physicians have a dual role in providing myriad supportive care as well, and their involvement can augment patient-centered communication and symptom management [36]. A retrospective chart review of 941 pediatric oncology patients found only 5% received palliative care consults despite more than a quarter of them having a poor prognosis [61]. Although previous indications for initiating a palliative care consult have been identified, they were typically not consulted until more than four indications were present [61]. Palliative/supportive care consultation is associated with more patient-aligned goals, less disease-related symptom burden, less caregiver burden, better quality of life, improved communication amongst various medical teams, improved physician-to-patient and physician-to-family communication, better coordination of care, and increased time between documentation of AD and time of death of patient [12,36,58,61,73,74,75]. Introducing the palliative care team early often emphasizes these benefits [12,62,76], and should be considered a key component in early and patient-centered ACP.

Furthermore, the presence of a multidisciplinary team inspires families’ confidence in the medical team [11]. Delayed palliative service is associated with an increased likelihood of death in the pediatric intensive care unit (PICU) as opposed to at home or in a hospice, an increased likelihood to receive cancer-directed therapy 30 days prior to death, and being less likely to have ADs in place at time of patient’s death [20,77]. One study did demonstrate that pediatric oncology providers with ≤10 years of experience reported the benefits of a multidisciplinary team more frequently than providers with more years of experience [78]. This could potentially be due to the recent establishment of pediatric palliative care within pediatric oncology, allowing more recent trainees to be more familiar with the service and their benefits.

In cases where the child’s death is inevitable, early discussions of ACP can aid in establishing a “good death”. This is a concept that is understudied in pediatrics, but essentially allows the EOL experience to be framed as a way of creating memories and honoring or celebrating the patient as per the family wishes [79]. Parents ranked “maintaining hope and pleasure” and “being respected as an individual” as the most important components of a good death [80]. Their perception of a good death was also most strongly associated with EOL plan discussion and parental agreement of establishing a living will [80]. Pediatric oncology healthcare providers have also reported communication, symptom management, and parental acceptance as vital factors of a good death [78].

Early conversations surrounding ADs and other aspects of ACP can include goals of care to be discussed before a potential medical crisis occurs, [16,33] as opposed to during the crisis, when parents have limited time to make decisions during an even more psychologically distressing experience. However, sometimes these situations are unavoidable. Emphasizing parental responsibility to the child rather than the parent’s personal desires or rights can minimize the emotional conflict that parents may feel during such intense decision-making, particularly if parents disagree with each other over the best decision for their child [16]. Disagreements can also arise between medical physicians and parents who may differ in opinions on optimal medical treatment in advanced cancer [24,81]. Refocusing ACP onto the patient in cases where physicians or parents disagree could allow for greater congruence in treatment decisions and eventually prevent more futile medical interventions down the road.

## 7. Conclusions

While early and patient-centered ACP in children with refractory oncological disease is important, there is no standardized, widely accepted protocol in guiding physicians on how and when to initiate ACP conversations with families and when to involve a palliative care team. Some intervention tools have been developed to implement early ACP with pediatric oncology patients; current literature shows positive results with implementation of any one of these tools but they require validation and comparison [26,27,28,33,53,66,70]. It is also important to consider the limitations of these interventions. Many of these tools either completely exclude non-English speaking families or only include 1–2 languages besides English in their screening questionnaires. Broadening the available languages in these tools can improve efficacy of information-sharing between physicians and families. Future studies are needed for younger and school-aged children regarding their direct involvement in ACP-related conversations, as most current interventions tools are targeted towards adolescents and young adults [11,15].

Children who are diagnosed with advanced cancer and their families undergo a significant emotional burden due to their illness, which families may experience for years after their child’s passing. Physicians can offer some guidance by introducing the concept of ACP early in the disease course to optimize congruence between patient, family, and the medical team and aid in navigating this difficult journey. Physicians have often delayed conversations due to apprehension over parental reactions, decreasing parents’ hope, and disease prognosis uncertainty. However, parents have demonstrated preference to have open and honest conversations as early as feasible, and these conversations have been shown to decrease their overall emotional burden. The evidence supports early initiation of these discussions, taking the time to sit with families and assess their goals and values and validate their concerns, acknowledging uncertainties but being transparent about prognosis where possible, encouraging openness in communication from all parties, providing resources as needed to parents who would like to have the conversation with their child, and providing additional training for providers. The incorporation of a palliative care team can help facilitate these conversations and assist in refocusing conversations on the patient. ACP has been demonstrated to be beneficial to patients and families in numerous ways and should be routinely considered in the pediatric oncology patient population.

## Supplementary Materials

The following supporting information can be downloaded at: https://www.mdpi.com/article/10.3390/children12040479/s1, Table S1: Parent-centered versus patient-centered patient care.
