# Supplementary material for: Advance Care Planning Conversations in Pediatric Patients with Refractory Oncologic Disease
Source: Children (Basel). 2025 Apr 8;12(4):479. doi: 10.3390/children12040479 (PMC12026240; doi:10.3390/children12040479)
Supplement: Supplementary file 1 [file children-12-00479-s001.zip › children-3539004-supplementary.pdf]

	Advantages	Disadvantages
Parent-Centered Care	• Protection of vulnerable pediatric patients<br>• Parental expertise and knowledge about their child	• Potential for conflicting interests versus patient's desires overlooked<br>• Associated with increased intensity of EOL care *
Patient-Centered Care	• Respect for patients' autonomy<br>• Greater semblance of patient control<br>• Increased patient understanding of illness<br>• May lead to greater adherence to and acceptance of treatment plan<br>• Increased congruence of treatment plan amongst patient, family, and medical team<br>• Decreased parental decisional regret<br>• Decreased depression/anxiety scores for patient and parents at follow-ups **<br>• Decreased parental uncertainty regarding their child's wishes<br>• Higher likelihood of ADs and other documentation in place<br>• Higher quality of EOL care	• May be difficult to ascertain level of appropriateness to involve patient<br>• Increased short-term emotional distress on family/patient in initiating difficult conversations<br>• Increased likelihood of legal/ethical dilemmas if disagreements between patient and family arise<br>• Patient's level of capacity likely to fluctuate through course of illness and requires frequent reassessment<br>• Time-consuming

Table S1. Comparison of Parent-Centered and Patient-Centered Care.

* Studies have not shown whether this is in line with patient's wishes, but when patient's wishes are factored in studies have shown it is associated with less intense EOL care

** Decreased parental decisional regret may persist for years beyond patient's death
